# Gut Microbiota Signatures Are Associated With Psychopathological Profiles in Patients With Ulcerative Colitis: Results From an Italian Tertiary IBD Center

**DOI:** 10.1093/ibd/izad091

**Published:** 2023-06-06

**Authors:** Franco Scaldaferri, Antonio Maria D’Onofrio, Rosaria Calia, Federica Di Vincenzo, Gaspare Filippo Ferrajoli, Valentina Petito, Eleonora Maggio, Pia Clara Pafundi, Daniele Napolitano, Letizia Masi, Elisa Schiavoni, Caterina Fanali, Pierluigi Puca, Laura Turchini, Loris Riccardo Lopetuso, Federica Del Chierico, Lorenza Putignani, Antonio Gasbarrini, and Giovanni Camardese

**Affiliations:** UOS Malattie Infiammatorie Croniche Intestinali, Centro di Malattie dell’Apparato Digerente (CEMAD), Fondazione Policlinico Universitario “A. Gemelli” IRCCS, Università Cattolica del Sacro Cuore, Rome, 00168, Italy; Dipartimento di Medicina e Chirurgia traslazionale, Università Cattolica del Sacro Cuore, Rome, 00168, Italy; Dipartimento di Neuroscienze, Sezione di Psichiatria, Università Cattolica del Sacro Cuore, Rome, 00168, Italy; Servizio di Psicologia Clinica, Fondazione Policlinico Universitario Agostino Gemelli IRCCS, Università Cattolica del Sacro Cuore, Rome, 00168, Italy; Divisione di Chirurgia Generale e del Trapianto di Fegato, Dipartimento di Scienze Mediche e Chirurgiche, Fondazione Policlinico Universitario Agostino Gemelli, IRCCS, Rome 00168, Italy; UOS Malattie Infiammatorie Croniche Intestinali, Centro di Malattie dell’Apparato Digerente (CEMAD), Fondazione Policlinico Universitario “A. Gemelli” IRCCS, Università Cattolica del Sacro Cuore, Rome, 00168, Italy; Dipartimento di Neuroscienze, Sezione di Psichiatria, Università Cattolica del Sacro Cuore, Rome, 00168, Italy; UOS Malattie Infiammatorie Croniche Intestinali, Centro di Malattie dell’Apparato Digerente (CEMAD), Fondazione Policlinico Universitario “A. Gemelli” IRCCS, Università Cattolica del Sacro Cuore, Rome, 00168, Italy; Servizio di Psicologia Clinica, Fondazione Policlinico Universitario Agostino Gemelli IRCCS, Università Cattolica del Sacro Cuore, Rome, 00168, Italy; Divisione di Chirurgia Generale e del Trapianto di Fegato, Dipartimento di Scienze Mediche e Chirurgiche, Fondazione Policlinico Universitario Agostino Gemelli, IRCCS, Rome 00168, Italy; Research Core Facility di Epidemiologia e Biostatistica, Gemelli Generator, Fondazione Policlinico Universitario Agostino Gemelli IRCCS, Rome, Italy; UOS Malattie Infiammatorie Croniche Intestinali, Centro di Malattie dell’Apparato Digerente (CEMAD), Fondazione Policlinico Universitario “A. Gemelli” IRCCS, Università Cattolica del Sacro Cuore, Rome, 00168, Italy; UOS Malattie Infiammatorie Croniche Intestinali, Centro di Malattie dell’Apparato Digerente (CEMAD), Fondazione Policlinico Universitario “A. Gemelli” IRCCS, Università Cattolica del Sacro Cuore, Rome, 00168, Italy; UOS Malattie Infiammatorie Croniche Intestinali, Centro di Malattie dell’Apparato Digerente (CEMAD), Fondazione Policlinico Universitario “A. Gemelli” IRCCS, Università Cattolica del Sacro Cuore, Rome, 00168, Italy; UOS Malattie Infiammatorie Croniche Intestinali, Centro di Malattie dell’Apparato Digerente (CEMAD), Fondazione Policlinico Universitario “A. Gemelli” IRCCS, Università Cattolica del Sacro Cuore, Rome, 00168, Italy; UOS Malattie Infiammatorie Croniche Intestinali, Centro di Malattie dell’Apparato Digerente (CEMAD), Fondazione Policlinico Universitario “A. Gemelli” IRCCS, Università Cattolica del Sacro Cuore, Rome, 00168, Italy; UOS Malattie Infiammatorie Croniche Intestinali, Centro di Malattie dell’Apparato Digerente (CEMAD), Fondazione Policlinico Universitario “A. Gemelli” IRCCS, Università Cattolica del Sacro Cuore, Rome, 00168, Italy; UOS Malattie Infiammatorie Croniche Intestinali, Centro di Malattie dell’Apparato Digerente (CEMAD), Fondazione Policlinico Universitario “A. Gemelli” IRCCS, Università Cattolica del Sacro Cuore, Rome, 00168, Italy; Immunology, Rheumatology and Infectious Diseases Research Area, Unit of Human Microbiome, Bambino Gesù Children's Hospital, IRCCS, Rome, Italy; Unit of Microbiology and Diagnostic Immunology, Unit of Microbiomics and Immunology, Rheumatology and Infectious Diseases Research Area, Unit of Human Microbiome, Bambino Gesù Children's Hospital, IRCCS, Rome, Italy; UOS Malattie Infiammatorie Croniche Intestinali, Centro di Malattie dell’Apparato Digerente (CEMAD), Fondazione Policlinico Universitario “A. Gemelli” IRCCS, Università Cattolica del Sacro Cuore, Rome, 00168, Italy; Dipartimento di Medicina e Chirurgia traslazionale, Università Cattolica del Sacro Cuore, Rome, 00168, Italy; Dipartimento di Neuroscienze, Sezione di Psichiatria, Università Cattolica del Sacro Cuore, Rome, 00168, Italy

**Keywords:** ulcerative colitis, behavioral disorders, gut microbiota, psychopathology

## Abstract

**Background:**

Several patients with ulcerative colitis (UC) suffer from psychiatric disorders, such as major depressive disorder, anxiety, or bipolar disorder, and show specific personality traits. Despite this, there are few data about personality profiles’ characterization in UC patients and about correlation of their psychopathological profile with their intestinal microbiota.

The aim of our study is to analyze the psychopathological and personality profile of UC patients and correlate it with specific signatures of their gut microbiota.

**Methods:**

This is a prospective interventional longitudinal cohort study. We enrolled consecutive patients affected by UC attending to the IBD Unit of Center for Digestive Disease of “A. Gemelli” IRCCS Hospital in Rome and a group of healthy subjects, matched for specific characteristics. Each patient was evaluated by a gastroenterologist and a psychiatrist. Moreover, all participants underwent psychological tests and a collection of stool samples.

**Results:**

We recruited 39 UC patients and 37 healthy subjects. Most patients showed high level of alexithymia, anxiety symptoms, depressive symptoms, as well as neuroticism and hypochondria, with obsessive-compulsive features at the behavioral level, which significantly impaired their quality of life and abilities at work. Gut microbiota analysis in UC patients demonstrated an increase in actinobacteria, Proteobacteria and Saccharibacteria (TM7), with a reduction in verrucomicrobia, euryarchaeota and tenericutes.

**Conclusions:**

Our study confirmed the presence of high levels of psycho-emotional distress in UC patients, alongside alterations of the intestinal microbiota, and highlighted some families and genera of bacteria (*Enterobacteriaceae*, *Streptococcus*, *Veillonella*, *Klebsiella,* and *Clostridiaceae*) as potential markers of an altered gut-brain axis in these patients.

**KEY MESSAGES**

Inflammatory bowel diseases (IBDs) are notoriously associated with psychiatric comorbidities, especially with anxiety and depression. Several studies demonstrated that they are consistent throughout IBD course and independent of disease activity. Despite this, literature is poor about characterization of psychological profiles of IBD patients. Therefore, our study aims to define the personality and psychopathological profile of UC patients and to correlate it with specific signatures of their gut microbiota. This could lead us to deepen our understanding of the gut-brain axis, which may be of help in the future to potentially develop “psychobiotis,” probiotics with a modulatory effect on the gut-brain axis.

## Introduction

By “gut-microbiota-brain axis,” we mean a dense network of connections that allow a 2-way communication between gut and the brain. Even though its exact function is still unknown, its role in keeping gastrointestinal and nervous system homeostasis is clear.^1^ The gut microbiota covers 10^13^ to 10^14^ microorganisms, including bacteria, viruses, fungi, protozoa and archaea, which encodes for more than 3 million genes. It is thus a dynamic system that changes throughout the human life. Although the exact number of species contained in the gut is not yet known, it has been estimated the presence of more than 1000 species and 7000 strains. The most represented phyla are Firmicutes (60% to 80%) and Bacteroidetes (20% to 40%), with the remaining amount consisting of Verrucomicrobia, Actinobacteria, and a lesser extent of Proteobacteria.^2^ Communication from the gut microbiota to the brain mainly occurs via microbial metabolites, such as short-chain fatty acids (SCFAs), secondary bile acids (2Bas), and tryptophan metabolites. Another way through which the gut microbiota and the brain communicate is through neuronal pathways involving the vagus nerve and/or spinal afferents. Communication also occurs from the brain towards the gut-microbiota. The central nervous system can affect the microbiota gut both directly, secreting endocrine mediators (for example, catecholamines) at the level of the intestinal lumen, which subsequently act at the level of microbial receptors, and indirectly, through the modulation of the intestinal environment by both branches of the autonomic nervous system (ANS).^3^ Several factors affect the activity of gut-microbiota-brain axis, and among these we remember diet, genetic and epigenetic heredity, mode of delivery, environment, drugs, exercise, and socioeconomic status. On the other hand, there are a series of behaviors affected by the gut-microbiota-brain axis, such as food intake, social interaction, cognitive behavior, stress, and fear.^4^

More and more researchers have been investigating the involvement of the gut-microbiota-brain axis in the etiopathogenesis of diseases. The gut-microbiota-brain axis is linked to various diseases, such as neurological, psychiatric, and neurodegenerative ones, and related to the extraintestinal manifestations of diseases such as inflammatory bowel disease (IBD) and inflammatory bowel syndrome (IBS).

Several studies show that the gut microbiota composition in patients with IBD is different from that of healthy subjects. Diversity is reduced by 25% compared with healthy controls, and in terms of composition, firmicutes and bacteroidetes are decreased, whereas Proteobacteria and actinomycetes are increased.^5^ Consistently with the reduced taxonomic diversity, there is also a reduced diversity in microbiota metabolites. Quantitative and qualitative alterations in metabolites can lead to, among other things, an alteration of the defense capabilities of the intestinal mucosa and immune system.^6^ Bacterial metabolites play an important role in providing feedback for the normal functioning of the epithelial barrier and immune cells. Some metabolites induce an immune response by altering the integrity of the intestinal barrier. Among the most studied metabolites we can find SCFAs, seemingly decreased in ulcerative colitis (UC) patients, which contributes to detrimental composition by modulating the colonic microbiota.^7^

Regarding depression, the gut-microbiota-brain axis is potentially involved in several mechanisms. It can modulate the release and efficacy of monoaminergic transmission, alter of the activity and function of the hypothalamus-pituitary-adrenal axis, activate inflammation and immune response, as well as regulate the abundance of brain-derived neurotrophic factor (BDNF).^8^ In IBD, the gut-microbiota-brain axis seems linked to the development of a mental disorder due to the underlying inflammatory activity. It has been shown that these psychological disorders were associated with the relapse of the disease.^9^ Of course, by providing further insights on the functioning of the gut-microbiota-brain axis, these researchers aspire to discover new therapies to better cope with diseases. An example is represented by the use of antibiotics, probiotics, prebiotics, and synbiotic in association with standard treatment in Parkinson’s disease,^10^ and probiotics in major depressive disorder.^11^

Several studies have explored the psychopathological component of IBD and the presence of personality patterns. According to a systematic review by Neuendorf et al, there is a 20% prevalence rate of anxiety and a 15% prevalence rate of depression in IBD patients.^12^ Furthermore, the onset of depression and anxiety often coincides with the relapse of the disease.^13^ A study by L.-T. Kao et al demonstrates how patients with IBD were more likely to have bipolar disorder than those without.^14^ In a review by Sajadinejad et al, it has been emphasized that several patients consider their personality as an essential factor in the development of the disease. Also, in the same review, neuroticism, perfectionism, and alexithymia have been shown as the most common personality traits in subjects with IBD. However, although various personality traits have been described in individuals with IBD, none particular personality trait can be considered specific to the disease.^15^

The aims of our study were to characterize the psychopathological profile in UC patients, analyze how it is related to the severity of their disease, analyze the qualitative and quantitative differences of gut microbiota in these patients, and describe the correlation between clinical, psychopathological characteristics, and changes in their intestinal microbiota.

## Materials and Methods

### Patients and Study Design

This is a prospective interventional longitudinal cohort study. We recruited consecutive patients affected by ulcerative colitis who were at least 20 years old and attending to the IBD Unit of CEMAD (Center for Digestive Disease) of “A. Gemelli” IRCCS Hospital in Rome; we also recruited a control group of healthy subjects, aged between 21 and 60 years old. Enrolled patients with a histologically confirmed diagnosis of UC underwent a clinical interview and a physical examination with the gastroenterologist, which included the collection of personal data, routine demographics, extent and duration of disease, disease activity assessments, previous biologic and immunosuppressive drugs, and concomitant medication. Patients were considered multifailure at biologic therapy if they failed at least 2 different biological drugs. A psychiatric interview was performed to identify either past or current psychiatric disorders. All participants also underwent psychological tests and a collection of stool samples for the gut microbiota analysis. All procedures complied with the informed consent administered to the patients that planned a complete assessment of their psychophysical distress. Ethical approval for this study was obtained from Gemelli’s Hospital’s ethical committee (ID1886/Prot. N. 0011626/18).

Furthermore, we explored any association between disease activity, Mayo score, therapy multifailure, and all psychometric parameters.

We divided patients into 3 groups according to their disease activity, based on full Mayo score^16^: remission was defined as full Mayo score less than 2, mild to moderate disease activity as full Mayo score between 2 and 6, and moderate to severe if above 6.

### Procedures and Questionnaires

Patients were evaluated by the full Mayo score, including both endoscopic and clinical Mayo score.

All patients underwent the following psychological tests:

- **Minnesota Multiphasic Personality Inventory-2 (MMPI-2),** the most widely used psychometric test for measuring adult psychopathology worldwide. It is a 567-item, true/false self-report measure of a person’s psychological state.^17^ It consists of 9 validity and 10 clinical scales, 31 clinical subscales, 15 content and 27 content component scales, 16 supplementary scales, and PSY-5 scales (*Personality Psychopathology Five)*.
- **State-Trait Anxiety Inventory—Form Y (STAI Y1 e Y2)**, a widely used self-report rating scale designed to measure 2 dimensions of anxiety, state anxiety (defined as a transitory feeling of tension and apprehension) and trait anxiety (relatively stable individual differences in anxiety proneness and general tendency to respond with anxiety to perceived threats in the environment).^18^ It is a 40-item scale, using a 4-point Likert scale for each item. It consists of 2 separated subscales, STAI-S for state anxiety and STAI-T for trait anxiety, each one consisting of 20 items.^19^
- **Hospital Anxiety and Depression Scale (HADS),** a questionnaire consisting of 14 items measuring symptom severity on a scale from 0 to 3, with subscales for anxiety (HADS-A) and depression (HADS-D), and a range of possible scores for each subscale of 0 to 21.^20^
- **Psychological General Well-Being Index (PGWBI)**, a validated health-related quality of life (HRQoL) measure providing a general evaluation of self-perceived psychological health and wellbeing. It consists of 22 self-administered items, rated on a 6-point scale, which assesses the psychological and general wellbeing of respondents in 6 HRQoL domains: anxiety, depressed mood, positive wellbeing, self-control, general health, and vitality. Each domain is defined by a minimum of 3 to a maximum of 5 items. The total score can reach a maximum of 110 points, which represent the best achievable “wellbeing.”^21^
- **General Self-Efficacy Scale (GSE)**, a measure of a person’s optimistic self-beliefs in coping with the demands of life. It consists of 10 items rated from 1 (“completely agree”) to 4 (“completely disagree”). The total score ranges from 10 to 40. A higher score indicates stronger self-efficacy.^22^
- **Connor-Davidson Resilience Scale (CD-RISC).** It contains 25 items, all measured on a 5-point scale (0, not true at all; 1, rarely true; 2, sometimes true; 3, often true; 4, true nearly all of the time). The scale is rated based on how the subject has felt over the past month. The total score ranges from 0 to 100, with higher scores reflecting greater resilience.^23^
- **Toronto Alexithymia Scale-20 (TAS-20)**, a self-report measure of alexithymia. It consists of 20 items, organized in 3 subscales: difficulty identifying feelings (7 items), difficulty describing feelings (5 items), and externally oriented thinking (8 items). A subject with a TAS total score ≥61 is considered to have alexithymia.^24^
- **Gastrointestinal Symptoms Rating Scale (GSCS).** It consists of 15 questions designed to assess the impact of upper and lower gastrointestinal symptoms; 5 subscales refer to reflux, diarrhea, constipation, abdominal pain, and indigestion. Each question produces a mean subscale score ranging from 0 (no discomfort) to 3 (very severe discomfort). Higher scores represent worse gastrointestinal symptoms.^25^

### 16S rRNA Targeted Metagenomics of Fecal Microbiota

According to the manufacturer’s instructions, DNA from stool samples was manually extracted using QIAmp Fast DNA Stool Mini Kit (Qiagen, Hilden, Germany). The 460-nucleotide (nt) variable region (V3-V4) from the 16S rRNA gene (Primer fw: 16S_F 5ʹ-TCG TCGGCAGCGTCAGATGTGTATAAGAGACAGCCTACGGGNGGCWGC AG)-3ʹ; primer rv: 16S_R 5ʹ (GTCTCGTGGGCTCGGAGATGTGTATAAGAGACAGGACTACHVGGGTATCTAATC C)-3ʹ was amplified by quantitative polymerase chain reaction (qPCR), for each sample, as described in the MiSeq rRNA Amplicon Sequencing protocol (Illumina, San Diego, CA).

The first PCR reaction was set up using the following conditions: one step at 95°C for 3 minutes, 32 cycles at 95°C for 30 seconds, at 55°C for 30 seconds, at 72°C for 30 seconds, and a final step at 72°C for 5 minutes. DNA amplicons were cleaned up by KAPA Pure Beads (Roche Diagnostics, Mannheim, Germany). Indexed libraries were obtained by using Nextera technology (Illumina). The final library was cleaned up using AMPure XP beads and quantified using Quant-iT™ PicoGreen dsDNA Assay Kit (Thermo Fisher Scientific, Waltham, MA).

According to the manufacturer’s specifications, samples were pooled together before the sequencing on an Illumina MiSeqTM platform (Illumina, San Diego, CA, United States) to generate paired-end reads of 300 base-length.

### Biocomputational and Statistical Analysis

Illumina Miseq reads were first analyzed for quality, length, and chimera presence using the Qiime v1.8 pipeline.^26^ Then, sequences were organized into operational taxonomic units (OTUs) with a 97% clustering threshold of pairwise identity. The PyNAST v.0.1 program was used to carry out a multiple sequence alignment against Greengenes 13_08 database, with a 97% similarity for bacterial sequences. Alpha diversity was performed by Qiime.

All statistical analyses were performed using SPSS software (Version 26.0. Armonk, NY: IBM Corp). The sample was described in its whole characteristics by descriptive statistical techniques. In depth, quantitative data were described by mean and standard deviation (SD) or median and interquartile range (IQR), as appropriate, whereas qualitative as absolute and relative percentage frequency. To verify Gaussian distribution of quantitative variables, the Shapiro-Wilk test was applied. The rate of UC patients with a score above the cutoff value on the different scales was reported as qualitative data. The correlation between the scores of each scale and gut microbiota was assessed by Pearson’s or Spearman’s correlation coefficient, as appropriate. Multivariate analysis of covariance (MANCOVA, covariates: age and disease activity) and Bonferroni post hoc analyses were performed to evaluate the quantitative differences of the different bacteria of gut microbiota between the patients with scores above or below the cutoff. The Mann-Withney *U* test was performed to evaluate the quantitative differences in intestinal microbiota between patients and controls. We applied Kruskal-Wallis test to assess differences in microbiota diversity according to disease activity. Microbiota diversity was assessed through Shannon index (the sum of the proportion of each species relative to the total number of species in the community under analysis accounts for both abundance and evenness) and Chao index (based upon the number of rare classes). Associations between disease activity, Mayo score and psychometric scales, and between disease activity, Mayo score and intestinal microbiota were also performed. The Mann-Whitney *U* test was further applied to assess the correlations between therapy multifailure and psychometric scales.

We analyzed the numerous differences found in gut microbiota of patients with clinically relevant psychopathological profile. In the Discussion section, we focus on the bacteria with a deviation from the mean values of abundance of at least ± 0.02.

## Results

### Descriptive Sociodemographic Data

We recruited 39 UC patients and 37 healthy controls. The male/female ratio was similar among the 2 groups (18:21 and 17:20, respectively). The mean age of the enrolled subjects was respectively 40.7 ± 15.2 among UC patients and 36.2 ± 14.5 among healthy controls (HCs).

In the UC subgroup, 10 patients were in remission, 18 had mild to moderate disease activity, and 11 patients had moderate to severe disease activity. Thirty-two patients were treated with biological agents (tumor necrosis factor [TNF] modulators or vedolizumab). In addition, 11 multifailure UC patients were observed.

### Clinical and Psychometric Data

**Figure 1.**
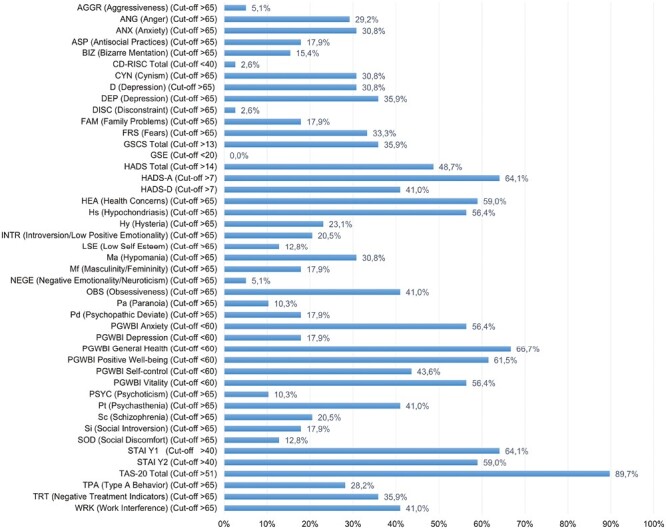
Psychometric data.

**Figure 2.**
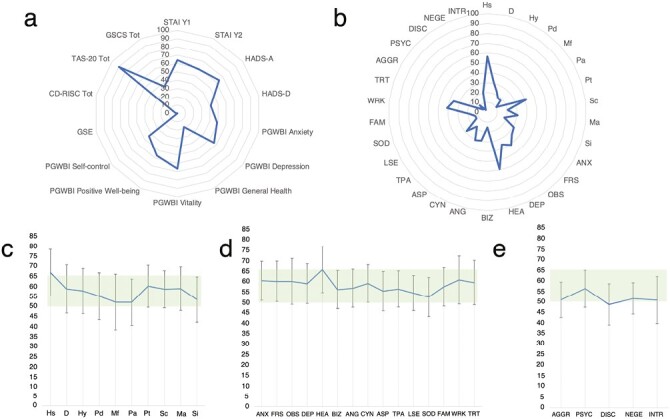
Psychometric data. A, Percentage of patients above the cutoff at the psychometrical scales. B, Percentage of patients above the cutoff at the MMPI-2 scales. C, Average ± SD of clinical scales (MMPI-2). D, Average ± SD of content scales (MMPI-2). E, Average ± SD of PSY-5 (MMPI-2).

The clinical data are summarized in Table 1. The psychometric data are presented in Figure 1. In Figure 2 we present the psychometric data on 2 spider plots and the average scores of each scale of the MMPI-2.

**Table 1.** Clinical data of UC patients.

	UC patients (N = 39)
Disease activity (*n*, %)	Remission (8; 20%) Mild-moderate disease activity (22, 56%) Moderate-severe disease activity (9, 23%)
Years of illness (yr ± SD)	9.9 ± 6.8
Therapy multifailure (*n*, %)	No (28, 71%) Yes (11, 28%)
Ongoing Biological Therapy/Former Biological Therapy	32/7
Concomitant steroid therapy (*n*, %)	No (15, 38%) Yes (5, 13%) Previous (19, 49%)

Abbreviations: Yr, years; SD, standard deviation

### Bacterial Abundance Between UC Patients and Healthy Controls

In our UC cohort, the phylum-level analysis demonstrated an increase in actinobacteria (*P* = .003), Proteobacteria (*P* = .010), and TM7 (*P* = .005), and a reduction in verrucomicrobia (*P* < .001), euryarchaeota (*P* < .001), and tenericutes (*P* < .001).

The family-level analysis demonstrated instead an increase in Aerococcaceae (*P* = .005), Gemellaceae (*P* < .001), and Planococcaceae (*P* < .001); and a reduction in Barniesellaceae (*P* < .001), Christensenellaceae (*P* < .001), Clostridiales incertae (*P* < .001), Mogibacteriaceae (*P* = .002), and Ruminococcaceae (*P* < .001).

**Figure 3.**
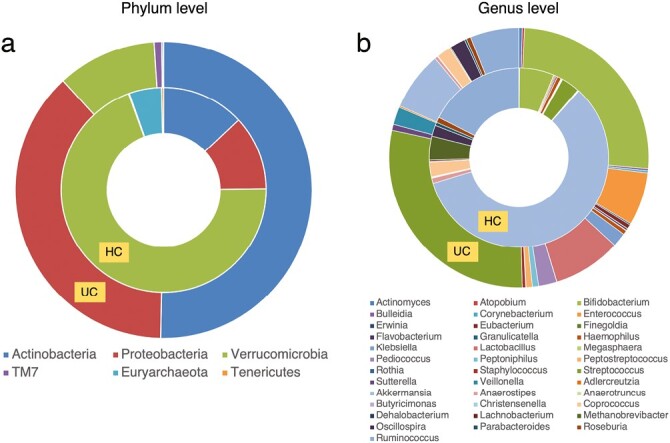
Bacterial abundance between UC patients and HC. A, Phylum-level analysis. B, Genus-level analysis. Abbreviations: HC, health control; UC, ulcerative colitis patients.

At genus-level analysis, we instead observed an increase in *Actinomyces* (*P* = .009), *Atopobium* (*P* = .001), *Bifidobacterium* (*P* = .006), *Bulleida* (*P* = .002), *Corynebacterium* (*P* = .017), *Enterococcus* (*P* < .001), *Erwinia* (*P* = .029), *Eubacterium* (*P* = .010), *Finegoldia* (*P* = .002), *Flavobacterium* (*P* < .001), *Granulicatella* (*P* = .002), *Haemophilus* (*P* = .001), *Klebsiella* (*P* = .015), *Lactobacillus* (*P* < .001), *Megasphaera* (*P* = .009), *Pediococcus* (*P* < .001), *Peptoniphilus* (*P* < .001), *Peptostreptococcus* (*P* = .001), *Rothia* (*P* = .002), *Staphylococcus* (*P* = .009), *Streptococcus* (*P* < .001), *Sutterella* (*P* = .006) and *Veillonella* (*P* < .001); a reduction in *Adlercreutzia* (*P* < .001), *Akkermansia* (*P* < .001), *Anaerostipes* (*P* < .001), *Anaerotruncus* (*P* = .018), *Butyricimonas* (*P* = .002), *Christensenella* (*P* = .005), *Coprococcus* (*P* = .016), *Dehalobacterium* (*P* < .001), *Lachnobacterium* (*P* = .021), *Methanobrevibacter* (*P* < .001), *Oscillospira* (*P* = .002), *Parabacteroides* (*P* = .032), *Roseburia* (*P* = .007) and *Ruminococcus* (*P* < .001; Figure 3).

**Figure 4.**
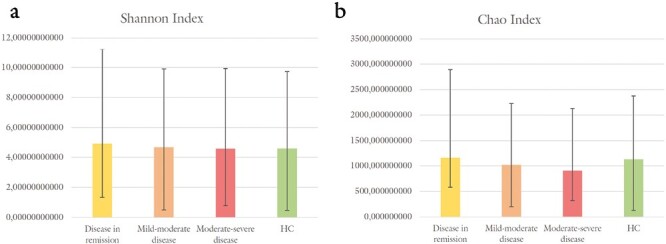
Bacterial diversity between UC patients grouped by disease severity and HC. A, Shannon Index. B, Chao Index.

We further assessed the Shannon index. We considered the control group and the 3 subgroups of UC patients. The highest Shannon index was observed in the UC group in remission (mean ± SD, 4.4 ± 1.59), with the lowest in the UC group with moderate to severe disease activity (mean ± SD, 4.57 ± 1.02). However, no significant difference was detected (Figure 4a). Likewise, for the Chao index, the highest was found in the UC group in remission (mean ± SD, 1154.13 ± 695.02), with the lowest among those with moderate to severe disease activity (mean ± SD, 904.82 ± 419.39), even in this case without any significant difference (Figure 4b).

### Correlations Between All Psychometric Scales and Intestinal Microbiota

In the Supplementary Materials, we show the correlation between psychometrical and MMPI-2 scales and microbiota in terms of Pearson’s correlation coefficients and related *P* value (Table S2 and Table S3 respectively, in Supplementary Materials).

Ulcerative colitis patients with a STAI Y1 score above the cutoff showed, at family level, a mild decrease in Aerococcaceae (*P* = .007), Enterobacteriaceae (*P* = .027), and Gemellaceae (*P* = .005); whereas at the genus level, there was an increase in *Acidaminococcus* (*P* < .001), *Actinomyces* (*P* = .019), *Bilophila* (*P* < .001), *Odoribacter* (*P* < .001), *Oribacterium* (*P* = .008), and a decrease in *Peptoniphilus* (*P* = .042), *Peptostreptococcus* (*P* < .001), *Sutterella* (*P* < .001), and *Veillonella* (*P* < .001). A STAI Y2 score above the cutoff instead disclosed, at the phylum level, a decrease in Actinobacteria (*P* = .030). At the family level, there was an increase in and Enterobacteriaceae (*P* = .037) and a decrease in Aerococcaceae (*P* = .014) and Coriobacteriaceae (P = .009); whilst at genus level, there was an increase in *Christensenella* (*P* = .022), *Flavobaterium* (*P* = .043), *Oribacterium* (*P* = .027), *Sutterella* (*P* = .005), and *Veillonella* (*P* = .009), and a decrease in *Adlercreutzia* (*P* < .001), *Atopobium* (*P* < .001), *Bilophila* (*P* = .019), *Bifidobacterium* (*P* = .046), *Bulleida* (*P* < .001), and *Peptoniphilus* (*P* = .003).

An HADS-A score above the cutoff disclosed instead at the phylum level an increase in TM7 (*P* = .035); at the family level, it showed an increase in Barnesiellaceae (*P* = .020) and Gemellaceae (*P* < .001) and a decrease in Clostridiaceae (*P* = .014) and Peptostreptococcaceae (*P* = .004); and at the genus level, there was an increase in *Sutterella* (*P* = .003) and *Veillonella* (*P* = .008) and a decrease in *Acidaminococcus* (*P* = .002), *Atopobium* (*P* = .009), *Bilophila* (*P* = .001), *Clostridium* (*P* = .004), *Epulopiscium* (*P* = .008), *Klebsiella* (*P* = .013), *Odoribacter* (*P* = .003), and *Rothia* (*P* = .028). For what concerned HADS-D, a score above the cutoff on HADS-D showed at the family level an increase in Enterococcaceae (*P* = .039) and Gemellaceae (*P* = .008) and a decrease in Clostridiaceae (*P* < .001) and Peptostreptococcaceae (*P* = .029); at the genus level, there was an increase in *Sutterella* (*P* < .001) and *Veillonella* (*P* < .001) and a decrease in *Anaerococcus* (*P* < .001), *Clostridium* (*P* = .003), *Coprobacillus* (*P* = .029), *Epulopiscium* (*P* = .021), and *Lactobacillus* (*P* = .001).

Ulcerative colitis patients who scored below the cutoff on PGWBI Anxiety showed instead at the phylum level a decrease in TM7 (*P* = .001); at the family level, there was an increase in Enterobacteriaceae (*P* = .001) and Rikenellaceae (*P* = .016) and a decrease in Aerococcaceae (*P* = .049), Gemellaceae (*P* = .009), and Planococcaceae (*P* = .037); and at the genus level, there was an increase in and *Peptostreptococcus* (*P* < .001) and a decrease in *Epulopiscium* (*P* = .040), *Granulicatella* (*P* = .011), *Oribacterium* (*P* = .002), *Peptoniphilus* (*P* = .007), and *Rothia* (*P* = .031). As for the PGWBI Depression scale above the cutoff, we observed at the phylum level an increase in TM7 (*P* < .001); at the family level, there was an increase in Clostridiaceae (*P* = .007), Gemellaceae (*P* < .001), and Planococcaceae (*P* = .034) and a decrease in in Aerococcaceae (*P* = .002). At the genus level, there was an increase in *Actinomyces* (*P* = .015), *Atopobium* (*P* < .001), *Lactococcus* (*P* = .004), *Oribacterium* (*P* = .032), *Peptoniphilus* (*P* = .021), *Peptostreptococcus* (*P* = .034), *Streptococcus* (*P* < .001), *Turicibacter* (*P* = .005), and *Veillonella* (*P* = .001).

A score above the cutoff on D (MMPI-2) showed at the family level an increase in Comamonadaceae (*P* = .017) and Planococcaceae (*P* = .018) and a decrease in Aerococcaceae (*P* = .005); and at the genus level, there was an increase in *Acinetobacter* (*P* = .022), *Corynebacterium* (*P* = .004), *Holdemania* (*P* = .039), *Propionibacterium* (*P* = .019), *Pseudomonas* (*P* = .020), *Rothia* (*P* = .010), *Staphylococcus* (*P* = .034), *Streptococcus* (*P* = .021), and *Turicibacter* (*P* < .001) and a decrease in *Dialister* (*P* = .013), *Erwinia* (*P* = .037), *Finegoldia* (*P* = .045), *Klebsiella* (*P* < .001), *Sutterella* (*P* = .045) and *Veillonella* (*P* = .001). An ANX (MMPI-2) above the cutoff instead disclosed at the phylum level an increase in Proteobacteria (*P* = .016) and a decrease in TM7 (*P* = .002); at the family level, there was an increase in Enterobacteriaceae (*P* < .001) and a decrease in Aerococcaceae (*P* < .001), Comamonadaceae (*P* = .002), and Planococcaceae (*P* = .011); and at the genus level, there was an increase in *Christensenella* (*P* = .015), *Citrobacter* (*P* = .030), *Sutterella* (*P* = .001), and *Veillonella* (*P* < .001) and a decrease in *Acinetobacter* (*P* = .005), *Actinomyces* (*P* = .006), *Atopobium* (*P* = .008), *ClostridiaceaeSMB53* (*P* = .003), *Coprobacillus* (*P* = .012), *Corynebacterium* (*P* = .001), *Enterococcus* (*P* = .018), *Flavobacterium* (*P* = .002), *Propionibacterium* (*P* = .001), *Pseudomonas* (*P* = .004), *Staphylococcus* (*P* = .003) and *Turicibacter* (*P* = .007).

**Figure 5.**
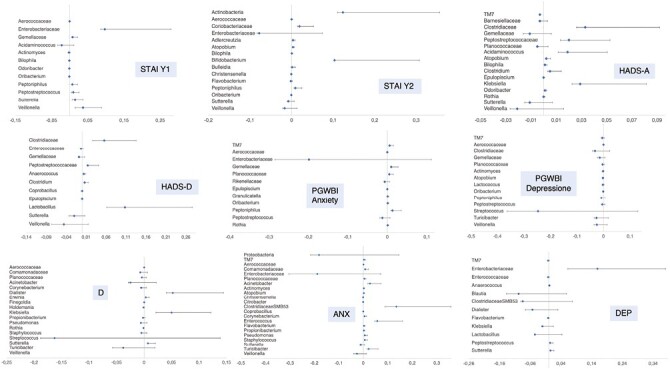
MANCOVA Analysis. The analysis shows the difference in mean bacterial abundance values between patients who did not overcome the cutoff on psychometric scales and those who did. The graphs show all the statistically significant differences detected: A negative value indicates a greater presence of the involved specific bacterium in the group of patients that exceeded the cutoff.

Finally, UC patients with a depression (DEP) (MMPI-2) score above the cutoff showed at the phylum level an increase in TM7 (*P* = .042); at the family level, there was an increase in Enterococcaceae (*P* = .011) and a decrease in Enterobacteriaceae (*P* < .001); and at the genus level, there was an increase in *Blautia* (*P* = .001), *ClostridiaceaeSMB53* (*P* = .034), *Dialister* (*P* = .001), *Flavobacterium* (*P* = .025), *Klebsiella* (*P* = .017), and *Lactobacillus* (*P* = .040) and a decrease in *Anaerococcus* (*P* = .001), *Peptostreptococcus* (*P* = .008), and *Sutterella* (*P* = .012; Figure 5).

**Figure 6.**
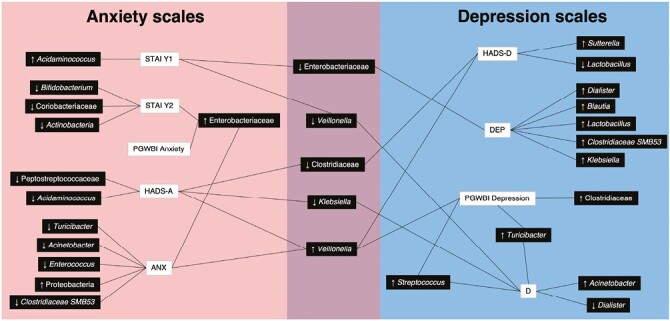
Bacteria associated with anxiety and depression scales.

In Figure 6 we summarize the bacteria associated with the anxiety and depression scales.

### Relationship Between Disease Activity and All Psychometrics Parameters

Patients with moderate to severe disease activity disclosed the lowest scores on the Difficulty Describing Feelings (DDF) factor scale of the Toronto Alexithymia Scale (TAS-20; mean ± DS, 13,27 ± 3,349; KW test, *P* = .041). No association was found with the other scales.

As for the Mayo score, we did find a significant correlation only with the endoscopic Mayo score but none with the clinical and full Mayo scores. Of note, we found higher scores on the “self-confidence and negative emotion management” subscale of the Connor-Davidson Resilience Scale (CD-RISC; mean ± DS, 22.50 ± 3.028; KW test, *P* = .019) and on the Total CD-RISC (mean ± DS, 79\.30 ± 11.557; KW test, *P* = .040) in patients with endoscopic Mayo score of 2 (moderate activity). We further observed that the score decreased in patients with an endoscopic Mayo score of 3 (severe activity; mean ± DS, 16.18 ± 4.513 and 62.09 ± 12.926, respectively).

**Figure 7.**
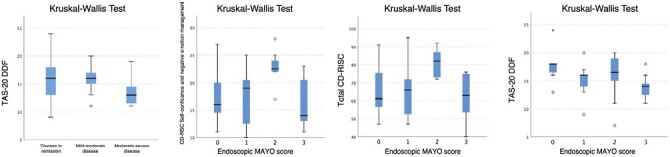
Correlations amongst clinical and endoscopic disease activity and all psychometrics parameters.

We also found higher scores on the Difficulty Describing Feelings factor scale of the TAS-20 (Toronto Alexithymia Scale; KW test, *P* = .038) in patients with endoscopic Mayo score 0 (mean ± DS, 17.71 ± 3.302) and lower scores in patients with Mayo score 3 (mean ± DS, 13.82 ± 1.991; Figure 7).

### Association Between Disease Activity and Intestinal Microbiota

When considering disease activity, we discovered that patients in remission had a greater quantity of *Coprococcus* genus (*P* = .019), patients with a mild to moderate disease activity had a greater quantity of *Anaerofustis* (*P* = .022), *Collinsella* (*P* = .011)*, Dialister* (*P* = .031)*, Lachnospira* (*P* = .007), and *Ruminococcus* (*P* = .013) genera, and patients with a moderate to severe disease activity had a greater quantity of phylum Proteobacteria (*P* = .15), family Enterobacteriaceae (*P* = .005), and genus *Lactobacillus* (*P* = .034).

Considering the Mayo score, we found significant correlations for clinical, endoscopic, and full Mayo scores. The correlation of clinical Mayo score and microbiota revealed that patients with Mayo clinical score 2 to 4 had higher levels of the genus *Ruminococcus* (*P* = .028); patients with clinical Mayo score 5-7 had higher levels of the phylum Proteobacteria (*P* = .035); and patients with clinical Mayo score >7 had higher levels of the phylum Tenericutes (*P* = .009) and the family Enterobacteriaceae (*P* = .014).

## Discussion

In this study, we deeply analyzed an essential feature of UC patients, represented by their psychopathological profiles, by comparing a cohort of UC patients and a control group matched for age and sex. Despite the crucial impact of this aspect on the development and the evolution of disease, literature is still scarce. Moreover, for the first time we investigated the correlation between the psychopathological profiles of UC patients and specific signatures in their gut microbiota.

When analyzing the scores on the psychometric scales of UC patients, it became clear that most of them had a high level of alexithymia (89.7% positive for TAS-20) and significantly relevant anxiety symptoms; a high percentage of patients who exceeded the cutoff on the STAI Y1, STAI Y2, and HADS-A scales were below the cutoff on the PGWBI anxiety subscale. Moreover, depressive symptoms, which were represented in a relevant part of the patients, did not seem to be negligible, as about 41.0% of the subjects affected by UC exceeded the cutoff on the HADS-D subscale. A significant percentage also had scores below the cutoff on the PGWBI General Health and PGWBI Positivity and Well-Being subscales, indicating that the quality of life of these subjects with UC is generally significantly impaired. On the other hand, scales examining resilience and self-efficacy were not particularly altered in these patients. The GSCS scale, which examines gastrointestinal symptoms, was instead elevated in 35.9% of the sample.

Analysis on the MMPI-2 test revealed a high percentage of patients with scores above the cutoff of 65 T-points in the following scales: Hypochondriasis (Hs), Psychasthenia (Pt), Obsessiveness (OBS), Depression (DEP), Health Concerns (HEA), Work Interference (WRK) and Negative Treatment Indicators (TRT). Among the basic scales, Hypochondriasis and Psychasthenia scales are the ones that exceeded most the cutoff of 65 T-scores. Therefore, we may assume that UC patients have polarized thoughts about the typical physical symptoms of their disease, which could be responsible for a steady state of hyperarousal and hypochondria. High Pt scores indicate anxious symptomatology accompanied by obsessive-compulsive traits, with a particular tendency of the subjects to ruminate about their own worries or to engage in compulsive, highly disabling rituals.

The presence of obsessive-compulsive symptoms was also confirmed by the OBS content scale. These are patients who tend to be perfectionists. It is likely that the obsessive aspect is used to cope with the pathology. They develop an obsession to control a medical situation from which they suffer. A great concern for their health can also be detected on the content scales (related to the hypochondria scale). There may be compromises in terms of treatment (TRT) and work (WRK). These patients are likely to have difficulty getting to work during the most acute phase of the illness and are unable to be fully productive. In terms of treatment, they have greater difficulty relying on introspective treatment, and they are therefore more likely to be on supportive therapy. These patients are less able to understand the disease on a psychological level, perceiving the disease only in the context of medical symptoms. The best treatment for them is a combined one, both pharmacological and supportive. In summary, these patients are characterized by neuroticism and hypochondria, with obsessive-compulsive features at the behavioral level, resulting in impaired ability to work and difficulties at the treatment level.

Our findings are consistent with the literature. In fact, a systematic review by Neuendorf et al reported that in patients with IBD, there is a prevalence of 20% of anxiety disorders and 15% of depressive disorders and, to a relatively lesser extent, the presence of obsessive-compulsive, phobic, panic, dysthymic, and agoraphobic disorders.^12^ In a study by Byrne et al, 30.3% of patients with IBD have anxiety and/or depression,^27^ and Leone et al found that obsessive-compulsive disorder was more common in patients with active disease. This could be related to the need to control the unpleasant and unpredictable clinical manifestations such as diarrhea and abdominal pain.^28^ Regarding the personality component, some studies have shown that neuroticism is the personality trait most pronounced in patients with IBD. Perfectionism and alexithymia are also present, but to a lesser extent.^15^

As for the distribution of bacterial taxa of the intestinal microbiota in patients compared with HCs, our findings are partially consistent with previous literature. As in our work, a study by Xu et al demonstrated that in the inflamed intestinal mucosa of IBD patients there was an increase in Proteobacteria and a decrease in Firmicutes.^29^ Previous studies confirmed our findings of a decrease in protective bacteria, which produce short-chain fatty acids, such as Ruminococcaceae and increase in pro-inflammatory bacteria, such as Enterobacteriaceae^30,31^ in IBD patients. Moreover, similar to several previous studies,^32–38^ we showed that in patients with UC there is an increase in Actinobacteria and *Streptococcus* and a decrease in *Bacteroidetes*, *Roseburia*, *Akkermansia muciniphila*, and *Ruminococcus gnavus*. Although the development of UC is not associated with a specific bacterium, it has been observed how different bacteria, some known and others unclassified, can affect the diversity of gut microbiota, leading to dysbiosis.^7^ The different events following one another and starting from intestinal dysbiosis can be summarized as follows: (1) alteration of the intestinal flora with the onset of dysbiosis; (2) impairment of the innate and adaptive immune system; (3) increased inflammatory response of the gut; (4) rapid increase of pathogenic bacteria in the gut; (5) release of enterotoxins, which increase the permeability of the gut, and immunosuppressive proteins, which lead to dysregulation of the immune system; (6) damage to the intestinal mucosa by pathogenic bacteria; (7) excessive growth of certain bacteria causing changes in energy metabolism, increased inflammation, and further damage to the mucosa; (8) translocation of pathogenic bacteria; and (9) further damage to the mucosa (ie, a vicious cycle is created).^32^

We further evaluated both Shannon and Chao diversity indices and found that in our UC sample, the diversity of the microbiota seemed reduced in patients with moderate to severe disease activity, although not significantly. The change most associated with UC is the reduction in the diversity of the fecal microbiota.^7^ This was confirmed in a study by Moayyedi et al which found that the stools of patients treated with fecal microbiota transplantation (FMT) had greater microbial diversity than those of patients treated with placebo.^39^ In another study by Chen et al Shannon and Chao indices improved in patients with UC treated with FMT, so that the remission of these patients could be related to the changes induced by the manipulation of the microbiota.^40^

We then analyzed the changes in the microbiota in patients who presented a clinically relevant psychopathological profile, and we decided to focus our attention on the scales describing anxious and depressive symptoms, which appeared significantly elevated in our UC sample—considering the potential effect of age and disease activity, since both can affect the composition of the microbiota.^41–43^

Our study showed that increases in Enterobacteriaceae correlated with increases in anxiety symptoms (high scores on the STAI Y2 scales, below the cutoff on the PGWBI anxiety subscale, and elevated scores on the ANX). Conversely, we noted a decrease of Enterobacteriaceae in subjects who exceeded the cutoff on the STAI Y1. Previous studies suggest that patients characterized by trait anxiety (ie, an anxiety that occurs over time and in different situations) will “constitutively” show an increase in Enterobacteriaceae compared with those characterized by state anxiety (ie, an anxiety that develops under specific conditions). These differences highlight the complexity of the study, as well as the interpretation of a correlation between a biological parameter and a psychopathological dimension divided into numerous components such as anxiety. Chen et al found an increase in Enterobacteriaceae (as well as *Escherichia-Shigella*) in patients with active generalized anxiety disorder compared with healthy controls. Moreover, these bacteria were positively related to the severity of anxiety.^44^ In our study, we found a statistically significant reduction in Enterobacteriaceae in subjects who exceeded the cutoff at the Depression subscale. Although in the literature the increase of Enterobacteriaceae tends to be associated with depressive symptomatology or major depressive disorder,^45,46^ we must keep in mind that there are several studies showing that a greater presence of these pathobionts can induce behavioral and psychological changes in both humans and animals.^47–50^ In interpreting this association, we must consider that the Enterobacteriaceae family produces SCFAs (such as acetic and formic acids) through the fermentation of carbohydrates, which induce the biosynthesis of serotonin at the level of enterochromaffin cells, which are the main producers of serotonin.^51^ It cannot be excluded that the reduction of Enterobacteriaceae entails a reduction in serotonin levels, which, according to the aminergic theory, seems to be reduced in depressed individuals.^52^ It is still unclear whether depressive symptoms are associated with either an increase or decrease in Enterobacteriaceae.

A systematic review by Simpson et al^53^ highlighted that in some studies^44,45,54,55^ there was a positive correlation with depressive symptoms and Enterobacteriaceae, whereas in another^56^ there was a lower frequency of this family in either depressed individuals or those with depressive symptoms. Further evidence of the association between Enterobacteriaceae and depressive symptoms comes from a study by Jiang et al who investigated the effects of antidepressant therapy on the microbiota. In this study, depressed subjects, compared with healthy controls, showed an increase in Enterobacteriaceae (as well as other bacteria)^45^ in response to antidepressant therapy.

The increase in *Streptococcus* in our sample was most strongly associated with depressive symptoms (scores below the cutoff on the PGWBI Depression subscale and high scores on D). Several systematic reviews (Simpson et al 2020, Barandaouzisi et al 2020, Cheung et al 2019) considering different studies found that the genus *Streptococcus* (along with other bacteria) increased in individuals with major depressive disorder.^53,57,58^*Streptococcus* (along with *Clostridium*, *Klebsiella, Parabacteroides, Oscillibacter*, and *Alistipes*) can be counted among the bacteria with a high capacity to metabolize amino acids and proteins. Increased metabolism of proteins by the microbiota leads to an increase in toxic products such as ammonia, putrescin, and phenol.^59^ In relation to depression, higher protein intake has been found as associated with higher severity of depressive symptoms.^60^ Despite the association with depressive symptoms, the genus *Streptococcus* (along with *Candida*, *Escherichia*, and *Enterococcus*) has been found to produce serotonin,^61^ somewhat calling into question their role in the development of depression.

The genus *Veillonella*, like Enterobacteriaceae, also appeared either increased or decreased based on the scale with which it was associated (decreased in those who scored high on the STAI Y1 and D, increased in those who scored high on the HADS-A, HADS-D, and ANX, and scores below the cutoff on the PGWBI Depression). As the increase/decrease is associated with both depressive and anxious symptoms, we cannot define or hypothesize in each case the role of *Veillonella* in the development of these symptoms. In different studies, *Veillonella* was found elevated (along with other bacteria) in individuals with depression.^45,55,62–64^*Veillonella* is one of the bacteria with lipopolysaccharides (LPSs) at the outer membrane level.^65^ Several studies^65–67^ have shown how LPS interacts with macrophages and stimulates the immune response by releasing pro-inflammatory cytokines. Indeed, depressed individuals have been found to have an increase in pro-inflammatory interleukins (ILs) such as IL-1 and IL-6 and a decrease in anti-inflammatory cytokines such as IL-4 and IL-10.^66,67^

Additionally, a reduction in *Klebsiella* was found to be associated with both anxious and depressive symptoms (HADS-A and D beyond the cutoff). Our findings in this case are not consistent with the literature. A study by Lin et al reported an increase in *Klebsiella* in subjects with depression. Such differences could be due to the use of different psychometric instruments to assess depressive symptoms. In our study, self-evaluation scales were used, whereas Lin et al assessed patients with depression using the diagnostic criteria for major depressive disorder from the Diagnostic and Statistical Manual of Mental Disorders (DSM) and the Hamilton D scale, a hetero evaluation scale.^68^

A reduction in Clostridiaceae was associated with both anxious and depressive symptoms (HADS-A and HADS-D beyond the cutoff). Our findings even in this case are not consistent with the literature. Chen et al found an increase in this bacterial family in individuals with depression.^56^ Again, we hypothesize that the differences in results may be due to the use of different psychometric instruments to assess depressive symptoms. As in the study by Lin et al, patients were assessed using the DSM and the Hamilton D scale.^68^

In recent years, the bidirectional interaction between the gut microbiota and central nervous system has been of increasing interest due to the harmful effects of dysbiosis on brain function. Dysbiosis has been associated with different psychiatric disorders such as depression, anxiety, bipolarism, psychosis, and schizophrenia, though no specific association has been detected. Depleted levels of *Faecalibacterium* and *Corprococcus* and enriched levels of *Eggerthella* are shared between several psychiatric disorders, suggesting these conditions are characterized by a reduction of anti-inflammatory SCFAs-producing bacteria and an increase of pro-inflammatory genera.^69^ A study by Jiang et al showed in patients with generalized anxiety disorders (GAD) a decrease of *Firmicutes spp*., especially *Butyricicoccus* and *Lachnospira*, and of the anti-inflammatory commensal bacterium *Faecalibacterium*, and an increase of Fusobacteria and *Bacteroidetes* spp.^54^ Another study revealed an enrichment in *Escherichia-Shigella* and *Bacteroides* in a Chinese population with GAD, highlighting the relationship between the presence of pathogens and anxiety.^44^ Up to now, several studies have found an imbalance of microbial communities in patients with GAD or other psychopathological disorders, hypothesizing that gut microbiota could exert a leading role in driving the development or affecting the evolution of these disorders. This effect is more likely exerted through the alteration of the biosynthesis and metabolism of neurotransmitters, such as serotonin, gamma aminobutyric acid, dopamine, and norepinephrine produced by bacteria like *Bifidobacterium*, *Lactobacillus acidophilus*, *Enterococcus*, *Escherichia Coli,* and *Streptococcus*. Nishida et al found that stress in medical students after final examination pressure significantly decreased *Bifidobacterium* and increased *Streptococcus*. These findings suggest that the gut microbiota and its ability to rapidly adapt to the variation of external factors could be the linking factor that determines the development of anxiety and depression following environmental leverages.^70^ Therefore, microbiome modulation through diet or probiotics may represent a preventive and therapeutic tool for psychiatric disorders.

Diet affects the composition and richness (alpha-diversity) of gut microbiota. For example, diet alterations with reduced ingestion of indigestible carbohydrates can generate an imbalance of microbial diversity and richness, reducing gut *Firmicutes* and increasing *Bacteroides* phyla. Moreover, alterations of the gut microbiota decrease the intake of calories from the diet, altering the immunological response.^71^

A *psychobiotic* can be defined as “live microorganisms that, when administered in adequate amounts, confer a health benefit on patients suffering from psychiatric diseases.” Several studies tested the efficacy of different probiotics, both with single and multiple strains of bacteria, for the reduction of anxiety and depression symptoms. Positive results for the significant reduction of stress and anxiety symptoms have been observed with the use of a single strain of *Bifidobacterium,* or multiple strains of *Lactobacillus*, especially *L. plantarum* and *L. rhamnosus*. Particularly, *L. plantarum P8* significantly decreased tumor necrosis factor (TNF)-α and interferon-gamma (IFN-γ) after treatment, whereas *L. plantarum DR7* significantly decreased cortisol levels, enhanced the serotonin pathway, and increased IL-10 levels.^72^ The analysis of the fecal metagenomes from the study with *L. plantarum P8* showed a significant increase in the prevalence of some species-level genome bins related to neuroprotective properties, such as *B. adolescentis*, *B. longum*, and *F. prausnitzii.*^72^ Additionally, *Faecalibacterium prausnitzii* demonstrated to increase SCFAs and IL-10 levels and reduce corticosterone and IL-6 levels in a rat model of mild stress.^73^ Probiotics resulted in more efficiently reducing anxiety in patients with either higher baseline anxiety or stress levels. Moreover, no significant difference was detected between the main outcomes and the use of single-strain or multistrain products, suggesting that the intrinsic characteristics of the strains and their combinations—and not the number of strains—determine their efficacy.^71^ Interestingly, in a murine model of colitis and chronic stress, *Weissella paramesenteroides* WpK4 showed their beneficial role in the gut-brain axis, determining a reduction of anxiety-like and depressive-like behaviors, through the reinforcement of the intestinal barrier and their immunomodulatory effect.^74^

Similarly, beneficial effects from the use of probiotics have also been observed in depression. A recent meta-analysis showed that the use of probiotics significantly improved the mood of patients with mild-moderate depressive symptoms compared with placebo.^75^*Bifidobacterium longum 1714* has been evaluated in 22 healthy volunteers under a cold-pressor test; the anxiety score (STAI) did not significantly increase under stress after one month of treatment, in contrast with the placebo.^76^ These findings suggest the possibility of using probiotics not only for treatment but also for the prevention of anxiety states. However, more studies are needed to corroborate this preventive effect.

Finally, we checked whether there was an association between disease activity and psychometric scales. Patients with moderate to severe disease activity had lower scores on the DDF factor scale of the TAS.20. This shows us that alexithymia is lower in these patients as the disease progresses. All of this could correlate in a congruent way with greater emotional vulnerability in these patients in the context of more severe pathology. The more the patient shows physical discomfort, the more he is able to perceive his psycho-emotional discomfort.

## Limitations of the Study

This study is not void of several limitations. First, the sample size of both patients and HC is relatively small. Plus, a single time point of the microbiota was assessed; the rest of the study follow-up is ongoing. There are no psychiatric patients, and therefore, an analysis of their microbiota is not available. Other data aimed at explaining the dysbiosis in the treatment of the disease, and the psychopathological profile of the patients should be seen in specific studies, appropriately evaluated. The assessment of anxiety and depression, although performed with different psychometric instruments, foresaw the use of self-evaluation scales. In the future, an assessment performed using hetero evaluation scales could be important.

## Conclusions and Future Perspectives

Our prospective, interventional, longitudinal cohort study confirms the presence of high levels of psycho-emotional distress in UC patients. This plays a role in treatment adherence and likely affects the clinical outcome to such an extent that a personalized and integrated “psycho-gastroenterological” approach is desirable to ensure the optimal outcome for each patient. Our study confirmed a significant difference between the microbiota of UC patients compared with controls, as reported in the literature. However, many variations in microbiota appeared to be significantly correlated with the expression of the patients’ psycho-emotional distress. Due to this, we believe that a concomitant rigorous analysis of the psychopathological profile of the patients is important for a correct evaluation and interpretation of the differences in the microbiota in the UC patients.

The data collected on the relationships between gut microbiota and psychopathological profile do not currently allow us to draw causal conclusions.

In a future perspective, it will be possible to deepen these correlations by using additional psychodiagnostic tools (including hetero evaluation) that can be administered to the same patient at different time points to be compared in turn with multiple evaluations of the microbiota itself in the context of the clinical course of each patient, with appropriate advance statistical methods. With a view to further investigation, these correlations between the psychopathological profile and the gut microbiota will also be examined in a psychiatric control sample to be compared with that of patients with UC.

However, at the end of this process we can say that some bacteria are potential markers of altered gut-brain axis in patients with UC, and of particular importance are the following: Enterobacteriaceae, *Streptococcus*, *Veillonella*, *Klebsiella*, and Clostridiaceae. These strains could be used for future studies to evaluate whether altering the microbiota could have an impact on this area and on disease. Strains suitable for probiotic development could be among the first examples of psychobiotics (probiotics that have a modulatory effect on the gut-brain axis).

## Supplementary Material

izad091_suppl_Supplementary_Material
