# Supplementary material for: Gut Microbiota Signatures Are Associated With Psychopathological Profiles in Patients With Ulcerative Colitis: Results From an Italian Tertiary IBD Center
Source: Inflamm Bowel Dis. 2023 Jun 6;29(11):1805–18. doi: 10.1093/ibd/izad091 (PMC10628916; doi:10.1093/ibd/izad091)
Supplement: izad091_suppl_Supplementary_Material [file izad091_suppl_supplementary_material.docx]

**SUPPLEMENTARY MATERIALS**

**Table 2** – Microbiota and psychometrical scale.

| **Microbiota** | **Psychometrical scale** | **Pearson's correlation coefficient** | ***P* value** |
| --- | --- | --- | --- |
| Proteobacteria | HADS Depression | 0.397 | 0.012 |
|  | HADS Total | 0.333 | 0.038 |
| Verrucomicrobia | PGWBI General Health | 0.333 | 0.039 |
| TM7 | PGWBI Depression | -0.436 | 0.006 |
| *Acinetobacter* | PGWBI Anxiety | -0.431 | 0.006 |
|  | PGWBI Vitality | -0.358 | 0.025 |
| *Actinomyces* | PGWBI Depression | -0.392 | 0.014 |
| Aerococcaceae | PGWBI Depression | -0.445 | 0.005 |
|  | PGWBI Self-control | -0.365 | 0.022 |
| *Akkermansia* | PGWBI General Health | 0.333 | 0.039 |
| *Atopobium* | PGWBI Depression | -0.570 | <0.001 |
| Barnesiellaceae | STAI Y 2 | -0.371 | 0.020 |
|  | PGWBI Positive Well-being | 0.333 | 0.039 |
|  | CD-RISC Total | 0.330 | 0.040 |
| *Bifidobacterium* | GSCS Total | 0.317 | 0.049 |
| *Catenibacterium* | GSE | 0.399 | 0.012 |
|  | AQ Total | -0.328 | 0.041 |
| Christensenellaceae | PGWBI Anxiety | -0.419 | 0.008 |
|  | PGWBI Vitality | -0.322 | 0.045 |
| Clostridiaceae | PGWBI Depression | -0.382 | 0.016 |
| ClostridiaceaeSMB53 | PGWBI Positive Well-being | 0.363 | 0.023 |
|  | GSE | 0.325 | 0.043 |
|  | AQ Total | -0.338 | 0.035 |
| *Collinsella* | BIS-11 Total | 0.377 | 0.018 |
| Comamonadaceae | PGWBI Anxiety | -0.437 | 0.005 |
|  | PGWBI Vitality | -0.369 | 0.021 |
| *Corynebacterium* | PGWBI Anxiety | -0.397 | 0.012 |
|  | PGWBI Vitality | -0.382 | 0.016 |
| Enterobacteriaceae | HADS Depression | 0.349 | 0.030 |
| Erysipelotrichaceae | TAS-20 Total | -0.388 | 0.015 |
| *Eubacterium* | CD-RISC Total | 0.440 | 0.005 |
| *Flavobacterium* | PGWBI Anxiety | -0.383 | 0.016 |
|  | PGWBI Vitality | -0.332 | 0.039 |
| Gemellaceae | GSCS Total | 0.335 | 0.037 |
| *Granulicatella* | PGWBI Depression | -0.370 | 0.020 |
| *Klebsiella* | GSE | 0.0394 | 0.013 |
|  | AQ Total | -0.384 | 0.016 |
| *Lactococcus* | PGWBI Depression | -0.326 | 0.043 |
| *Odoribacter* | PGWBI Self-control | 0.332 | 0.039 |
|  | GSE | 0.343 | 0.033 |
| *Oscillospira* | STAI Y 2 | -0.375 | 0.019 |
|  | HADS Anxiety | -0.338 | 0.036 |
|  | HADS Depression | -0.332 | 0.039 |
|  | HADS Total | -0.373 | 0.019 |
|  | PGWBI Self-control | 0.351 | 0.028 |
|  | CS-RISC Total | 0.357 | 0.026 |
| *Parvimonas* | PGWBI Depression | -0.318 | 0.049 |
| Peptostreptococcaceae | GSE | 0.349 | 0.030 |
| *Peptrostreptococcus* | AQ Total | -0.348 | 0.030 |
|  | BIS-11 Total | -0.319 | 0.048 |
|  | TAS-20 Total | -0.406 | 0.010 |
| *Phascolarctobacterium* | STAI Y 1 | -0.342 | 0.033 |
|  | STAI Y 2 | -0.415 | 0.009 |
|  | GSCS Total | -0.324 | 0.044 |
| *Propionibacterium* | PGWBI Anxiety | -0.438 | 0.005 |
|  | PGWBI Vitality | -0.371 | 0.020 |
| *Pseudomonas* | PGWBI Vitality | -0.352 | 0.028 |
| *Staphylococcus* | PGWBI Anxiety | -0.418 | 0.008 |
|  | PGWBI Vitality | -0.341 | 0.034 |
| *Streptococcus* | PGWBI Depression | -0.358 | 0.025 |
| *Sutterella* | GSCS Total | 0.536 | <0.001 |
| *Veillonella* | GSCS Total | 0.490 | 0.002 |

**Table 3** – Microbiota and MMPI-2 scale.

| **Microbiota** | **MMPI-2 scale** | **Pearson's correlation coefficient** | ***P* value** |
| --- | --- | --- | --- |
| *Proteobacteria* | NEGE | 0.392 | 0.014 |
| *Acidaminococcus* | Hs | -0.383 | 0.016 |
|  | FRS | -0.401 | 0.012 |
|  | WRK | -0.338 | 0.035 |
| *Acinetobacter* | LSE | 0.341 | 0.033 |
| *Anaerofustis* | Hy | -0.334 | 0.038 |
| *Anaerotruncus* | Hs | -0.331 | 0.040 |
|  | HEA | -0.349 | 0.029 |
| *Bacteroides* | Si | -0.364 | 0.023 |
|  | SOD | -0.316 | 0.050 |
|  | AGGR | 0.329 | 0.041 |
|  | INTR | -0.316 | 0.050 |
| Barnesiellaceae | Mf | 0.443 | 0.005 |
| *Bilophila* | FRS | -0.423 | 0.007 |
| *Blautia* | Pd | 0.382 | 0.016 |
|  | Pt | 0.353 | 0.028 |
|  | Sc | 0.365 | 0.023 |
|  | FRS | 0.351 | 0.028 |
|  | DEP | 0.351 | 0.028 |
| *Butyricimonas* | Mf | 0.348 | 0.030 |
| Clostridiaceae | Hs | 0.344 | 0.032 |
| Clostridiaceae SMB 53 | DISC | 0.361 | 0.024 |
| *Collinsella* | Hs | 0.322 | 0.045 |
|  | Hy | 0.333 | 0.038 |
|  | Pa | 0.321 | 0.046 |
|  | Pt | 0.469 | 0.003 |
|  | Sc | 0.503 | 0.001 |
|  | Si | 0.390 | 0.014 |
|  | ANX | 0.442 | 0.005 |
|  | DEP | 0.355 | 0.027 |
|  | BIZ | 0.319 | 0.048 |
|  | LSE | 0.329 | 0.041 |
|  | WRK | 0.416 | 0.008 |
|  | TRT | 0.364 | 0.023 |
|  | NEGE | 0.320 | 0.047 |
|  | INTR | 0.376 | 0.018 |
| Comamonadaceae | LSE | 0.344 | 0.032 |
| Coriobacteriaceae | TPA | 0.385 | 0.015 |
| *Dialister* | Pa | 0.518 | 0.001 |
|  | ANG | 0.399 | 0.012 |
|  | PSYC | 0.421 | 0.008 |
| *Dorea* | Hs | 0.317 | 0.049 |
|  | Sc | 0.355 | 0.026 |
|  | DEP | 0.319 | 0.048 |
|  | BIZ | 0.334 | 0.038 |
| Enterobacteriaceae | D | 0.366 | 0.022 |
|  | NEGE | 0.347 | 0.030 |
| *Epulopiscium* | CYN | -0.354 | 0.027 |
|  | SOD | 0.322 | 0.045 |
| *Erwinia* | AGGR | 0.357 | 0.026 |
| *Eubacterium* | FAM | 0.406 | 0.010 |
| *Faecalibacterium* | Mf | 0.320 | 0.047 |
|  | Pt | -0.327 | 0.042 |
|  | ANX | -0.326 | 0.043 |
| Gemellaceae | BIZ | -0.407 | 0.010 |
|  | CYN | -0.316 | 0.050 |
|  | ASP | -0.325 | 0.044 |
|  | PSYC | -0.341 | 0.033 |
| *Lachnobacterium* | Mf | 0.360 | 0.025 |
|  | ASP | 0.343 | 0.032 |
| *Lactococcus* | DISC | 0.324 | 0.044 |
| *Odoribacter* | Hs | -0.435 | 0.006 |
|  | Si | -0.389 | 0.014 |
|  | HEA | -0.390 | 0.014 |
|  | SOD | -0.340 | 0.034 |
|  | WRK | -0.318 | 0.049 |
|  | AGGR | 0.357 | 0.026 |
|  | NEGE | -0.339 | 0.034 |
| *Oribacterium* | SOD | 0.342 | 0.033 |
| *Paraprevotella* | Mf | 0.339 | 0.035 |
| *Pediococcus* | ASP | 0.319 | 0.048 |
| *Peptoniphilus* | Hy | 0.366 | 0.022 |
|  | BIZ | -0.325 | 0.044 |
|  | PSYC | -0.390 | 0.014 |
| Peptostreptococcaceae | ASP | 0.354 | 0.027 |
| *Peptostreptococcus* | Pt | -0.343 | 0.033 |
|  | Sc | -0.489 | 0.033 |
|  | Ma | -0.346 | 0.031 |
|  | FRS | -0.365 | 0.022 |
|  | OBS | -0.345 | 0.032 |
|  | DEP | -0.367 | 0.022 |
|  | BIZ | -0.374 | 0.019 |
|  | ASP | -0.374 | 0.019 |
|  | SOD | -0.446 | 0.004 |
|  | WRK | -0.332 | 0.039 |
|  | TRT | -0.448 | 0.004 |
|  | PSYC | -0.377 | 0.018 |
| *Phascolarctobacterium* | Mf | 0.327 | 0.042 |
| *Propionibacterium* | LSE | 0.348 | 0.030 |
| *Pseudomonas* | LSE | 0.339 | 0.035 |
| *Roseburia* | D | 0.330 | 0.040 |
| *Rothia* | Hy | 0.372 | 0.020 |
| Ruminococcaceae | HEA | -0.335 | 0.037 |
| *Slakia* | PSYC | -0.333 | 0.039 |
| *Staphilococcus* | LSE | 0.345 | 0.031 |
| *Sutterella* | Pt | -0.333 | 0.038 |
|  | Sc | -0.375 | 0.019 |
|  | ANX | -0.340 | 0.034 |
|  | FRS | -0.395 | 0.013 |
|  | OBS | -0.320 | 0.047 |
|  | DEP | -0.337 | 0.036 |
|  | BIZ | -0.361 | 0.024 |
| *Turicibacter* | SOD | 0.357 | 0.026 |
|  | FAM | 0.325 | 0.043 |
